# A Dramatic Clinical Response to Trastuzumab-Deruxtecan in a Patient with HER-2 Low Breast Cancer with Untreated Leptomeningeal Metastasis and Hydrocephalus

**DOI:** 10.3390/curroncol32020081

**Published:** 2025-01-31

**Authors:** Sarah Hussain, Robert Nordal, Danny Ng, Morgan Willson, Xiaolan Feng

**Affiliations:** 1 Arthur Child Comprehensive Cancer Center, Calgary, AB T2N 5G2, Canada; sarah.hussain@albertahealthservices.ca (S.H.); robert.nordal@albertahealthservices.ca (R.N.); 2 Department of Radiology, Foothills Medical Center, Calgary, AB T2N 2T9, Canada; morgan.willson@albertahealthservices.ca; 3 Department of Radiation Oncology, Tom Baker Cancer Center, Calgary, AB T2N 4N2, Canada; danny.ng@albertahealthservices.ca

**Keywords:** HER2 low, trastuzumab-deruxtecan, leptomeningeal metastasis, hydrocephalus

## Abstract

Leptomeningeal metastasis (LM) is a rare and challenging manifestation of advanced breast cancer (ABC) with severe morbidity and mortality. Patients with LM may be asymptomatic, or present with non-specific neurologic deficits, thereby possibly delaying diagnosis. Treatment typically requires a multimodal approach for effective management, symptom relief, and quality-of-life improvement. Trastuzumab-deruxtecan (T-DXd), a humanized monoclonal antibody drug conjugate, demonstrated efficacy across diverse breast cancer subtypes expressing variable levels of HER2 proteins. Currently, T-DXd is the standard of care for patients with advanced, pretreated, HER2 low breast cancer. There is limited evidence of the response of brain metastases (BM) and leptomeningeal metastases (LM) to T-DXd in HER2-low patients, with most data extrapolated from HER2-positive breast cancer studies. This case report presents the first documented instance of a patient with debilitating, symptomatic, untreated LM and hydrocephalus demonstrating a rapid and dramatic clinical response to T-DXd. This finding holds crucial clinical relevance, highlighting the potential benefit of initiating effective systemic therapy for LM early in treatment to address both central nervous system (CNS) and non-CNS disease burden, rather than delaying systemic therapy until after radiation therapy.

## 1. Introduction

Leptomeningeal metastasis (LM) is a rare site of metastasis in breast cancer, with an almost 5% prevalence, and tends to occur late in the course of disease [1]. The reported median time of development is around 7.4 years from the onset of initial breast cancer diagnosis, and 21 months from metastatic disease [1]. It is associated with a poor outcome and dismal prognosis, with an estimated median overall survival (OS) time of 4.9 months [2]. Certain breast cancer biologic subtypes are shown to have high prevalence of LM compared to others.

For instance, infiltrating lobular carcinoma and HER2 positive breast neoplasms have high predilections to metastasis to the leptomeninges [3]. HER2 low breast cancer includes those who have an immunohistochemistry (IHC) score of 1+ and 2+ without amplification measured by an in situ hybridization (ISH) test. The rate of HER2 low breast cancer is around 40% to 62% in hormone receptor (HR) positive tumors, and less than 40% in HR-negative tumors [4]. The rate of HER2 low expression appears to increase with estrogen receptor (ER) high expression [5]. The clinical manifestation of LM differs depending on the site of leptomeningeal invasion. Notably, headache is one of the most common initially presenting symptoms, seen in 30% to 50% of cases [6].

Hydrocephalus is a common association with LM, and diagnosis requires a high index of suspicion. Hydrocephalus is usually communicating, but obstructive hydrocephalus can occur with bulky brain metastasis (BM) or metastasis causing cerebrospinal fluid (CSF) flow obstructions [7].

There is no gold standard diagnosis for LM, but magnetic resonance imaging (MRI) and CSF cytology are the most frequently used modalities. MRI has an estimated sensitivity and specificity of 75% and 77%, respectively, in the assessment of brain and spine involvement [8]. The most sensitive test for LM diagnosis is the identification of malignant cells in the CSF, reaching up to 90% [8].

While radiation therapy has been used standardly for LM in breast cancer, the toxicity of radiotherapy to the entire cranial and spinal CSF compartment limits the application of this treatment to better performing patients who are well enough to benefit from a treatment that often causes significant fatigue, lowered blood counts, and initial worsening of presenting CNS symptoms [9].

Surgical management of leptomeningeal disease is limited, and is often used to aid administration of more efficacious treatment via intrathecal delivery [10]. Its main role is to alleviate the symptoms of complicated hydrocephalus, rather than treatment of the underlying disease [10].

There is limited high-quality evidence to guide the optimal combination of radiation therapy with CNS penetrating systemic therapy for the treatment of LM, especially in the era of evolving modern systemic therapies [10].

Conventional chemotherapies used either intravenous (IV) or intrathecal (IT) administration, but there has been always a pharmacologic challenge for drug delivery beyond the blood–brain barrier with IV administration, and IT therapies have been of limited value and are not widely used.

Options to consider in the absence of targeted therapeutic options would include either high-dose IV methotrexate, or single-agent capecitabine [11,12]. Other options with some proven responses include etoposide, cisplatin, bevacizumab, vinorelbine, gemcitabine, and 5-FU [13].

There is limited prospective evidence to support the use of endocrine treatments, such as tamoxifen, aromatase inhibitors (AI), and cyclin-dependent kinase (CDK) 4/6 inhibitors in BM. The majority of available evidence comes from retrospective studies. Although these treatments demonstrate potential clinical activity and favorable toxicity profiles, they are not currently recognized as a standard of care [14,15,16].

There is paucity of data regarding systematic therapeutic options for LM, and most treatment approaches are extrapolated from treatment of active BM. The existing literature and guidelines efforts for BM are felt to be valuable and applicable for LM, as both BM and LM constitute the spectrum of metastatic cancer in the CNS.

T-DXd has demonstrated CNS effectiveness in HER2 positive breast cancer patients. However, data on its efficacy in HER2 low breast cancer with BM and LM are limited.

In this case report, we have demonstrated that effectiveness of initial treatment with T-DXd in a patient with HER2 low metastatic breast cancer presenting with symptomatic LM and complicated obstructive hydrocephalus.

## 2. Case Presentation

A 65-year-old female patient, with a known case of dyslipidemia, which is not treated with medication, and otherwise healthy. She was diagnosed with stage IIIB invasive lobular carcinoma of the left breast in April 2006 (Table 1). She underwent upfront left modified radical mastectomy and left axillary lymph node (ALN) dissection. Postoperative pathology revealed a tumor size of 1.5 cm in the breast (pT1), and 18 out of 20 ALN were positive for disease (pN2). Metastatic workup was negative (M0). The tumor was of Grade 2, ER 6/8, PR 7/8, and HER2 by immunohistochemistry was not expressed.

She received adjuvant chemotherapy in the form of epirubicin combined with cyclophosphamide for four cycles, followed by four cycles of docetaxel. Subsequently, she completed adjuvant radiotherapy to the left chest wall and ALN region (5000 cGy in 25 fractions and 4482.5 cGy in 25 fractions, respectively).

Adjuvant endocrine therapy (ET) with Tamoxifen was given from January 2007 until January 2012. Attempts with AI were unsuccessful because of severe arthralgia, and AI therapy was discontinued a few months later, during the same year of 2012.

The patient was under active surveillance until March 2018, when she presented with abdominal pain. She underwent a CT scan of the chest, abdomen, and pelvis, showing a large pelvic mass of 14.5 cm causing hydronephrosis, and no other sites of disease involvement. A bone scan was negative. Although there is no consensus guideline with regard to cytoreductive surgery in the advanced setting, our local breast tumor board agreed to attempt local debulking in her situation. She underwent extensive surgical resection of the gross peritoneal metastatic disease, hysterectomy, and bilateral-salpingo-oophorectomy in May 2018. Postoperative pathology revealed the recurrent tumor was consistent with breast origin, lobular in nature, ER > 66%, progesterone receptor (PR) 34% to 66%, HER2 IHC of +2, with a negative silver in situ hybridization (SISH) result.

In June 2018, she started first line cell CDK 4/6 inhibitor (Palbociclib) concomitant with endocrine therapy aromatase inhibitor (Letrozole). Later, she was switched to exemestane because of intolerance to letrozole. Successive CT scans during follow up showed complete radiologic response.

In April 2022, the patient developed new evidence of metastasis with new progressive infiltrative soft tissue process involving the retroperitoneum and peritoneum, as well as multiple new sclerotic bone lesions in the thoracic and lumbar spine. The patient’s treatment was switched from palbociclib and exemestane to capecitabine chemotherapy. She had side effects, including diarrhea and hand and foot syndrome, which required a dose reduction of chemotherapy. Upon further disease progression in July 2023, treatment was switched to weekly paclitaxel. Private genetic testing was discussed with the patient, but did not proceed due to a lack of funding for the targeted drug. T-DXd was not funded in our cancer program through any means for metastatic HER2 low breast cancer at that time.

**Figure 1:**
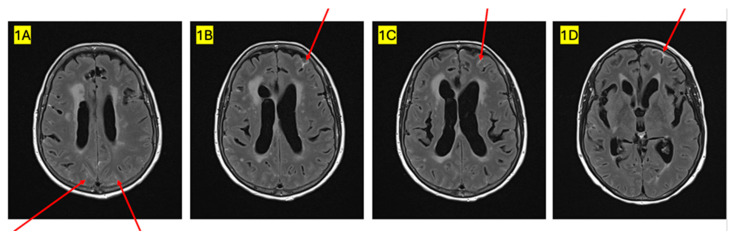
MRI FLAIR image demonstrating leptomeningeal enhancement within the parietal lobes bilaterally (**1A**), and multiple areas of leptomeningeal enhancement within the left frontal lobe (**1B**–**1D**).

**Figure 2:**
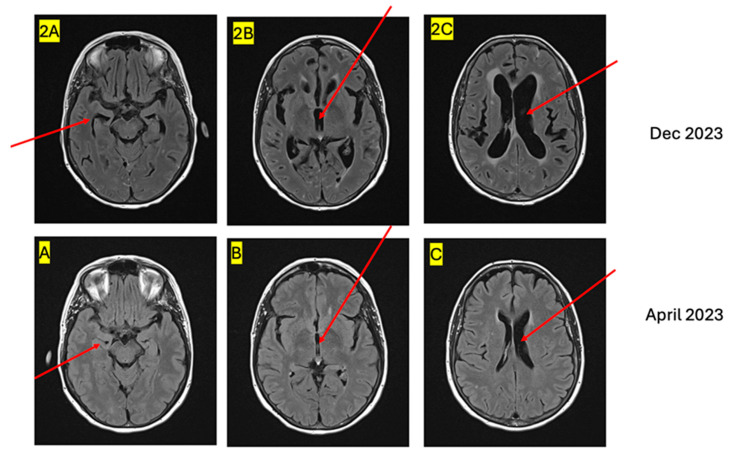
MRI FLAIR image demonstrating severity of hydrocephalus. (**A**,**2A**) Comparing the caliber of temporal horns; (**B**,**2B**) comparing caliber of the third ventricle; (**C**,**2C**) comparing caliber of the lateral ventricles).

In early December 2023, the patient reported a new onset headache. A brain MRI performed showed a new leptomeningeal disease complicated by hydrocephalus (Figure 1 and Figure 2). A spine MRI showed LM disease, which was seen in the lumbar region with diffuse enhancement of the cauda equina. About a week later, the patient presented with a worsening headache associated with morning nausea and right-sided weakness.

Both neurosurgery and radiation oncology services were consulted immediately after the scan. T-DXd was obtained through a compassionate access program at the same time. Surgical intervention for CSF flow management was only felt warranted if she became more symptomatic.

A computer-optimized volumetric modulated arc therapy (VMAT) radiation treatment plan was developed to deliver 3600 cGy to the neuroaxis in 20 daily treatment fractions, with partial vertebral marrow sparing, and sparing of head and neck, chest, and abdominal organs. Delivery of radiotherapy was held, however, after discussion within the oncology team and with the patient, with a preference for potentially applying CNS active systemic therapy first.

During the same time, T-DXd was initiated on 4 January 2024, through a compassionate access program. After the second cycle, the patient showed a dramatic clinical response with complete resolution of the headache and improvement of the right-sided weakness. After an extensive discussion, the patient and radiation oncology team mutually decided to hold off radiation therapy, and to continue T-DXd. She tolerated treatment well, apart from fatigue lasting 1 week post-treatment. She did not report any respiratory symptoms, such as a new cough or shortness of breath. Her performance status had significantly improved from 2–3 pretreatment to 0–1 after two cycles of T-DXd. Most importantly, her quality of life also significantly improved. She was able to continue physical activity as previously.

After third cycles of T-DXd, on March of 2024, a planned contrast enhanced brain, spine, and abdomen/pelvis MRI and non-contrast high resolution CT (HRCT) chest were performed in March 2024. Unfortunately, the MRI contrast was not given due to patient refusal. Although the MRI report showed an apparent improvement in LM disease and stable mild hydrocephalus, this was somewhat inconclusive due to the lack of a contrast-enhanced MRI. Her systemic disease including retroperitoneal and bone metastases were stable.

HRCT chest revealed bilateral ground glass opacities/infiltrates consistent with drug-induced interstitial lung disease (ILD). Upon further questioning, the patient admitted that she did have a new cough and shortness of breath, most noticeable with exertion post cycle 2 treatment. T-DXd therapy was discontinued after cycle 3, and high dose prednisone was initiated to manage ILD.

The duration of clinical response to T-DXd lasted around three months from the time of treatment initiation. Unfortunately, her neurological symptoms quickly returned and worsened after T-DXd discontinuation (Table 1). Radiation treatment was recommended, but she declined and was not well enough for further therapy. Approval for further systemic treatment with Sacituzumab govetican was obtained through a compassionate access program, but was unfortunately not given due to poor performance status. The patient was sent to hospice, and passed away on the 10 May 2024.

## 3. Discussion

The mechanism by which T-DXd exerts its function in patients with BM and LM is not clear. It is a dogma that large biologics such as T-DXd cannot cross the blood–brain barrier. Preclinical data suggested an increased deruxtecan payload measured inside the brain, and induction of tumor cell apoptosis in orthotopic models was confirmed by testing cleaved caspase 3 levels [13].

In the era of new advancements in HER2 low subtype breast cancer, T-DXd has shown clinical efficacy in this subcategory of patients. This is the first reported case of HR positive HER2 low ABC with untreated symptomatic LM and hydrocephalus showing a clinical response to T-DXd.

The first approval of T-DXd in HER2 low breast cancer came from the Destiny 04 clinic trial. In the subgroup analysis of patients with stable and treated BM (around 35 patients) the intracranial progression free survival (PFS) was 9.7 months vs. not evaluable due to small patient numbers, favoring T-DXd compared to the physician’s choice of chemotherapy. T-DXd was associated with ORR of 25%, CR around 16.7%, and the majority of patients (approximately 50%) had stable disease and no progression while on therapy [17].

Clinical data demonstrating the brain activity of T-DXd have largely come from HER-2 positive breast cancer clinical trials. Exploratory pooled analysis from the Destiny 01, 02, and 03 trials showed strong intracranial activity with T-DXd for both stable and active BM. The median PFS was around 12.3 months for T-DXd, compared to 8.7 months for the physician’s choice of systemic therapy in patients with treated and stable BM [18,19,20,21]. Interestingly, there was a noticeably greater advantage of T-DXd in untreated or active BM, with a median PFS reaching 18.5 months. The duration of intracranial response (ICR) was around 12.3 months for patients with treated/stable BM vs. 17.5 months for untreated or active BM [22].

The latest breaking primary results of phase III/IV Destiny-Breast 12 clinical trial presented in the ESMO Congress 2024. Patients with HER-2 positive breast cancer with BM had a durable CNS PFS of around 58.9% at 12 months. The results were similar to those with stable and active BM [23].

However, the prospective data to support the intracranial activity of T-DXd in HER2 low disease is relatively small, especially for patients with LM. DAISY was a phase II clinical trial with a cohort that included 10 patients with asymptomatic and treated HER2-low BM, where 7 patients were HR positive. The best objective response was 30%, clinical benefit ratio of 50%, and the median PFS was 4.1 months [24].

Several other additional small studies, such as TUXEDO-1 (*n* = 15) and case series from the DUKE cancer institute and Dana-Farber cancer center (*n* = 8), showed around 73% ICR to T-DXd [3,25,26]. In the most recent ongoing phase II trial (The DEBBRAH trial), one cohort of patients included both HER-2 + and HER-2 low breast cancer patients presenting with asymptomatic and untreated BM showed ORR- intracranial (IC) of 50%. ORR-IC was 44.4% in the cohort of the HER-2 + who had prior local brain therapy including surgery, stereotactic radiosurgery/stereotactic radiotherapy (SRS/SRT) or whole brain radiotherapy (WBRT). The preliminary reported results of cohort 5 were reported at SABCS 2023. That cohort included a total of seven patients with untreated HER2+ or HER2 low ABC and untreated leptomeningeal carcinomatosis. Four out of seven patients included in the cohort had HER-2 low disease. The median PFS in all patients included was around 8.9 months, and the median OS was 13.3 months. The majority of patients had prolonged stabilization for more than 24 weeks, and none experienced intracranial disease progression [27].

Few reported phase II clinical trials included patients with LM. We have the preliminary results of the ongoing (ROSET-BM) trial, which is investigating the clinical efficacy of T-DXd in HER-2 positive breast cancer with BM and LM. The results show that the 12 month PFS and OS rates were 60.7% and 87.1%, respectively [28].

One of the major toxicities of T-DXd is drug-induced pneumonitis, which is one of the leading causes of drug discontinuation. The incidence of drug-related pneumonitis in T-DXd clinical trials was around 15.4%. The majority of cases were Grade 1 and 2. As per ASCO 2023 recommendations, patients with Grade 2 T-DXd-induced pneumonitis should be treated with a systemic steroid and permanently discontinue T-DXd [29].

To the best of our knowledge, there is only case report on T-DXd therapy in HER2 low breast cancer with LM, published in November 2023: a 44-year-old woman with heavily pretreated HR positive HER2 low breast cancer and disease in the bone, liver, pleura, brain, and leptomeningeal disease. A brain MRI was performed because of dermatome V2 and V3 insult. She was treated with T-DXd, and continued for 17 cycles of therapy. Local therapeutic intervention to the brain was not reported. The patient experienced a partial repose to BM, significant decrease in meningeal thickness, and resolved neurologic symptoms [30].

The uniqueness of our case report is that T-DXd demonstrated dramatic and rapid response in a metastatic HR positive, HER2 low breast cancer patient with LM complicated by hydrocephalus who had debilitating neurologic symptoms on presentation, and had not yet received any local regional treatment such as surgical intervention or radiation treatment. This experience addresses paucity of both clinical trial and real-world data to support the use of T-DXd in this specific scenario.

The short treatment duration of T-DXd limited our ability to assess the potential durability of the response in LM and HER2 low patients. The duration of the initial marked clinical and radiological response in LM remains uncertain, particularly regarding how long it would persist if T-DXd treatment had been continued. It is very unfortunate that our patient did not have the chance to receive proton craniospinal irradiation (pCSI) due to rapid deteriorating performance status upon discontinuation of T-DXd.

There is growing evidence about the role of pCSI in breast cancer and non-small cell lung cancer (NSCLC). The results are promising, with significant improvement in CNS PFS and OS in comparison to the standard photon involved-field radiotherapy (IFRT) with a CNS median PFS of around 5.8 months and a median CNS OS of 6.6 months [31].

Initial craniospinal irradiation (CSI) radiation therapy can still be considered standard, but drawbacks include delays in systemic therapy to address the ongoing need for control of a non-CNS disease, and significant side effects of CSI, including headache and worsening CNS symptoms, fatigue, GI toxicity, and myelosuppression. Subsequent somnolence and negative cognitive effects are also of concern. While myelosuppression can be lessened with proton radiation therapy [31], or computer optimized VMAT radiation planning [32], the overall side effect burden of CSI remains significant, and recovery can impair the ability to proceed to effective systemic therapy.

Our case provides a basis for both optimism and caution in using CNS active systemic therapy as initial therapy for LM, versus traditional approaches using radiotherapy, such as CSI.

There is clearly a need for better understanding of best combination of CNS active systemic therapy and radiation therapy. Our case suggests that very close follow up with a contrast MRI should be employed at a frequency of 8 weeks or less. Optimal use of both therapies, including future consideration of concurrent therapy, may provide a more effective therapy for this challenging problem.

Future efforts are needed to explore the use of initial CNS active systemic therapy, with CSI or WBRT applied quickly at first sign of progression, or in the absence of a response to systemic therapy. It is hoped that some patients will have a pronounced initial response like our patient, allowing the use of systemic therapy first that addresses all body disease burdens, while also lessening the neuroaxis tumor burden for better and longer effectiveness of subsequent CNS irradiation.

## 4. Conclusions

There is paucity of data to support treating metastatic HR positive, HER2 low ABC with symptomatic LM with T-DXd prior to locoregional treatment. Our unique case report demonstrated a dramatic and rapid, albeit short-lived, clinical response to T-DXd in this scenario, consistent with a few other patients treated on trials and real-world practice.

Further randomized clinical trials are required to confirm if T-DXd or another ADC should be considered as a standard of care alternative, or as a sequential option in this subcategory of rare and challenging symptomatic LMs.

## Figures and Tables

**Table 1:** Timeline of events.

2006	2018	2022
Stage IIIB, ER+/PR+/HER-2(-ve)	Peritoneal metastasis ER+/PR+/HER-2(-ve)	Multiple new bone metastasis
Up front surgery Adjuvant CT (EC-T) Adjuvant Tamoxifen	Surgical debulking CDK 4/6 inhibitor (Palbociclib)+AI	Capecitabine chemotherapy
2023 (July)	2023 (December)	2024 (March)
New liver metastasis and significant increase in retroperitoneal disease	Leptomeningeal metastasis causing hydrocephalus	Disease progression and worsening Neurologic symptoms
Weekly paclitaxel	T-DXd	No further therapy

CT: chemotherapy, EC-T: epirubicin/cyclophosphamide followed by docetaxel chemotherapy, AI: aromatase inhibitor, T-DXd: trastuzumab-deruxtecan.

## Data Availability

The data are available in the electronic database and health records of the hospital. The access is limited by hospital policy.

## Abbreviations

The following abbreviations are used in this manuscript:

ABC	Advanced breast cancer
ADC	Antibody drug conjugate
AI	Aromatase inhibitor
ALN	Axillary lymph nodes
BM	Brain metastasis
CDK 4/6 inhibitor	Cyclin dependent kinase 4/6 inhibitor
CNS	Central nervous system
CR	Complete response
CSF	Cerebrospinal fluid
CSI	Craniospinal irradiation
ER	Estrogen receptor
HER2	Human epidermal growth factor receptor 2
HR	Hormone receptor
HRCT	High resolution computed tomography
IC	Intracranial
ICR	Intracranial response rate
IHC	Immunohistochemistry
IV	Intravenous
IT	Intrathecal
LM	Leptomeningeal metastasis
MRI	Magnetic resonance imaging
ORR	Overall response rate
pCSI	Proton craniospinal irradiation
PFS	Progression free survival
PR	Progesterone receptor
SISH	Silver in situ hybridization
SRS	Stereotactic radiosurgery
SRT	Stereotactic radiotherapy
T-DXd	Trastuzumab-deruxtecan
WBRT	Whole brain radiotherapy
